# Short-Term Impact of Scleral Lens Wear on Intraocular Pressure and Retinal Nerve Fiber Layer Thickness

**DOI:** 10.3390/life16071094

**Published:** 2026-06-30

**Authors:** Pabita Dhungel, Muteb K. Alanazi, Patrick Caroline, Lorne Yudcovitch, Maria Liu

**Affiliations:** 1 School of Optometry, University of California, Berkeley, CA 94720, USA; marialiu@berkeley.edu; 2 Optometry Department, College of Applied Medical Sciences, King Saud University, Riyadh 11362, Saudi Arabia; mkalanazi@ksu.edu; 3 College of Optometry, Pacific University, Forest Grove, OR 97116, USA

**Keywords:** scleral lens, intraocular pressure, retinal fiber thickness, contact lens

## Abstract

**Purpose:** To investigate the short-term impact of scleral lens wear on intraocular pressure (IOP) and retinal nerve fiber layer (RNFL) thickness. We hypothesized that scleral lens wear would produce a measurable elevation in IOP accompanied by detectable RNFL thinning compared with soft contact lens wear. **Methods:** This prospective, randomized, contralateral-eye crossover study included 31 healthy participants (mean age: 26 ± 3 years). Each participant wore a 16.5 mm scleral lens over one eye and a soft contact lens over the fellow eye for 8 h, with assignments reversed between visits. IOP was measured using two tonometers: a transpalpebral Diaton tonometer and a non-contact tonometer (NCT), and RNFL thickness was measured by optical coherence tomography at four time points: pre- and post-lens application, and pre- and post-lens removal. **Results:** Eyes fitted with scleral lenses exhibited a significant IOP increase immediately after lens application (pre-application: 11 ± 3 mmHg vs. post-application: 16 ± 4 mmHg, *p* < 0.001), sustained throughout 8 h of wear (pre-removal: 16 ± 4 mmHg), and returned to baseline after removal (11 ± 3 mmHg). No significant IOP changes were observed in soft contact lens-wearing eyes (*p* > 0.05). Scleral lens wear was also associated with small but statistically significant peripapillary RNFL thinning (pre-application: 110 ± 11 µm vs. post-application: 107 ± 11 µm, *p* < 0.001), which returned to baseline after lens removal. No significant RNFL changes were observed with soft contact lens wear (*p* > 0.05). Bland–Altman analysis revealed poor agreement between Diaton and NCT measurements, consistent with the published literature on transpalpebral tonometry. **Conclusions:** Short-term scleral lens wear was associated with transient IOP elevation and peripapillary RNFL thinning, both reversible upon lens removal, in healthy young adults. These findings highlight the need for further longitudinal investigation in at-risk populations such as those with ocular hypertension, keratoconus, or early glaucoma before clinical monitoring recommendations can be established.

## 1. Introduction

Scleral contact lenses have gained significant popularity over the last two decades due to their advantages in enhancing visual outcomes and comfort across a range of ocular conditions [1]. These lenses have demonstrated therapeutic potential by successfully fitting most patients with distorted corneas who are intolerant to other forms of vision correction, such as piggyback, hybrid, or corneal gas-permeable lenses [2]. Scleral lenses are essential in optimizing visual outcomes, protecting the ocular surface, improving ocular aesthetics, and supporting performance in athletic activities [3]. Corneal ectasia remains the primary indication for scleral lens fitting, followed by post-penetrating keratoplasty patients [2,4]. Scleral lenses have been shown to reduce higher-order aberrations, including coma and spherical aberrations, in corneal ectasias and surface irregularities [5]. Furthermore, severe ocular surface disease has long been an indication for large scleral lens use [6]. Patients suffering from exposure keratitis and ocular surface disease may particularly benefit from scleral lenses due to the fluid reservoir maintained behind the lens [7]. Scleral lenses have also proven useful in managing various eyelid conditions such as ptosis, eyelid coloboma, entropion, and ectropion [8]. Studies have documented a marked reduction in photophobia in 75% of patients treated with scleral lenses [9]. Despite the successes of scleral lenses, adverse events, such as an associated increase in intraocular pressure (IOP), have raised concerns [10]. The fitting of scleral lenses differs substantially from that of soft contact lenses. Scleral lenses typically vault over the cornea and rest on the adjacent bulbar conjunctiva. Mechanical pressure from eyelid blinking is hypothesized to displace scleral lenses deeper into the conjunctiva, generating a suction effect that may impact ocular structures such as the episcleral veins, trabecular meshwork, and associated drainage pathways [11]. The amount of posterior-directed force may contribute to resistance in aqueous humor outflow from the eye [12].

Although transient IOP elevations in healthy eyes may be self-limiting due to intact autoregulatory mechanisms, their clinical relevance should not be dismissed. Elevated IOP contributes to glaucomatous damage through two primary mechanisms: mechanical compression of optic nerve axons and vascular dysfunction resulting in ischemia to the optic nerve head [13]. Repeated or sustained IOP spikes, even in otherwise healthy individuals, have been recognized as important risk factors, with experimental evidence on animals demonstrating that repetitive IOP spikes cause retinal ganglion cell dysfunction and death, effects not observed with steady ocular hypertension of comparable magnitude [14]. This is particularly relevant in the context of scleral lens wear, where daily lens use may expose the eye to repeated pressure fluctuations over months or years. Furthermore, a subset of scleral lens wearers includes individuals with keratoconus, high myopia, or ocular surface disease populations that may carry an elevated baseline susceptibility to IOP-related structural damage due to compromised corneal biomechanics and altered optic nerve head morphology [15,16].

All current clinical methods for measuring IOP require direct contact with the cornea, such as with a Goldmann tonometer or using a non-contact tonometer on a bare corneal surface. Both methods present challenges when measuring IOP with scleral lenses in place [17]. Researchers have developed strategies to address this issue by measuring IOP immediately after lens removal and by obtaining measurements. In contrast, the scleral lens is still worn while a transpalpebral tonometer is used [18]. Several recent studies have examined the changes in lens settling time, fluid reservoir thickness, and IOP associated with scleral lens wear [6,18,19,20]. The majority of these studies reported an increase in IOP with scleral lens use. Some studies have reported no change in IOP after 2 h of scleral lens wear compared with fellow eyes that did not wear contact lenses [19,21]. Similarly, some studies have even observed decreases in IOP following lens wear, consistent with a normal diurnal fluctuation in IOP [22].

However, considerable heterogeneity exists across studies in terms of scleral lens types, sizes, and IOP measurement techniques, making direct comparisons challenging. Retinal nerve fiber layer (RNFL) defects are a structural manifestation of the damage caused by elevated IOP, such as in glaucoma, and typically precede the development of glaucomatous vision loss [23,24]. Therefore, elevated IOP raises concerns about potential damage to the RNFL.

RNFL thickness, measured by optical coherence tomography (OCT), is among the most sensitive structural markers of optic nerve integrity and has been widely validated as an early indicator of glaucomatous damage. However, it is important to acknowledge that OCT-based RNFL measurements are subject to inherent test–retest variability, reported values ranging from approximately 4.5 µm for global average RNFL to 5.8–8.1 µm at the quadrant level in healthy subjects, depending on the device and operator [25]. Additionally, RNFL thickness exhibits known short-term physiological fluctuations, including diurnal variation with greater thinning observed in afternoon and evening measurements compared to morning baselines [26]. These sources of variability must be considered when interpreting small but statistically significant RNFL changes and were accounted for in this study through standardized measurement timing and repeated scan averaging.

Several OCT-based studies have examined the structural effects of scleral lens wear on the optic nerve head. Walker et al. (2020) assessed global minimum rim width (MRW) as an indirect structural marker during scleral lens wear in 26 healthy adults and reported modest MRW thinning in both test and control eyes after six hours, attributing this primarily to diurnal variation rather than lens-induced IOP elevation [21]. Samaha and Michaud (2020 evaluated BMO-MRW in 20 healthy young adults during scleral lens wear and reported a statistically significant decrease of −8 µm after six hours, potentially attributable to IOP changes [27]. More recently, Michaud et al. (2025) extended this investigation to a keratoconus population and similarly reported significant BMO-MRW thinning correlating with IOP fluctuations during six hours of scleral lens wear [10]. Demirtaş et al. (2025) found no significant RNFL or ganglion cell–inner plexiform layer changes in 28 keratoconus patients following only 30–75 min of scleral lens wear, though this brief adaptation period limits comparability with extended wear studies [28]. Collectively, these studies have focused on optic nerve head morphological parameters, specifically MRW and BMO-MRW, while peripapillary RNFL thickness, a well-established and sensitive marker of optic nerve axonal integrity, has not been directly examined in the context of scleral lens-associated IOP changes over extended wear periods.

Studies have demonstrated that in glaucoma progressing eyes, each 1 mmHg higher average IOP during follow-up was associated with an additional average loss of 0.20 µm/year, and higher IOP levels were associated with faster RNFL loss over time, as measured by SD-OCT [29]. Despite growing evidence linking scleral lens wear to transient IOP elevation, the structural consequences of these pressure changes on the optic nerve, particularly regarding short-term RNFL integrity, remain largely unexplored. Existing studies have focused primarily on IOP measurement methodology and lens-settling dynamics, leaving a critical gap in our understanding of whether transient IOP increases during scleral lens wear are sufficient to induce measurable structural changes at the optic nerve head. Based on previously reported mechanisms of scleral lens-induced IOP elevation, including compression of episcleral veins and impairment of aqueous humor outflow, we hypothesized that short-term scleral lens wear would produce a measurable and statistically significant increase in IOP compared to soft contact lens wear, and that this IOP elevation would be accompanied by detectable peripapillary RNFL thinning as a structural correlate of transient optic nerve head stress [12,20]. This study aims to examine the short-term impact of scleral lens wear on IOP and RNFL thickness compared with soft contact lens wear in healthy young adults.

## 2. Methods

This randomized crossover study complied with the tenets of the Declaration of Helsinki and received approval from the Institutional Review Board at Pacific University College of Optometry. Randomization was carried out using an online randomization tool, and participants were assigned a sequential study identification number in the order of enrollment. The subjects were recruited through voluntary sign-up in response to the email sent via the school’s listserv for optometry and vision science students of various years. Written informed consent was obtained from all participants before the study commenced.

### 2.1. Subjects

A total of thirty-one healthy participants aged 18 to 40 years were recruited from the optometry and vision science program at Pacific University. All participants had a best-corrected visual acuity of 0.3 log MAR or better and blood pressure within the range of 90/60 to 135/90 mmHg in a normal resting position. Participants with any ocular pathologies, elevated intraocular pressure (IOP) greater than 20 mmHg at the screening visit, a history of glaucoma, or previous refractive surgery were excluded from the study. Subjects were instructed to refrain from hard physical activities and/or caffeine, marijuana, and/or alcohol consumption 6 h before all visits in the study, as these behaviors may influence the IOP.

Healthy participants were intentionally selected for this initial investigation to establish a physiological baseline free from confounding ocular pathology. This approach is consistent with prior scleral lens IOP and optic nerve head studies that similarly recruited healthy adults to isolate lens-induced effects from disease-related variability [20,21]. Findings from this healthy cohort are intended to serve as a normative reference against which future studies in clinical scleral lens populations, such as those with keratoconus, corneal ectasia, or ocular surface disease, can be compared.

### 2.2. Measurements and Data Collection Procedures

**Figure 1:**
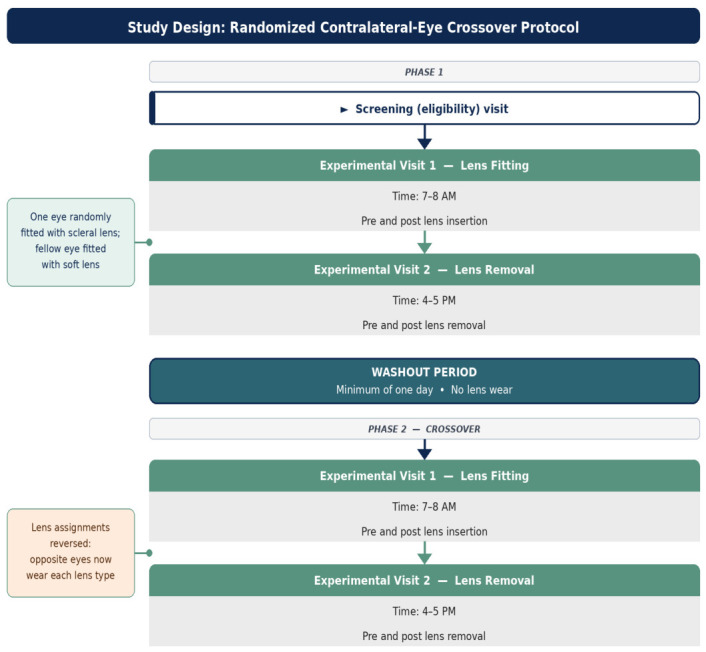
Flowchart illustrating the study data collection procedures in screening and experimental visits.

This study required 5 visits over 3 days (Figure 1). The Baseline day’s visits include scleral contact lens determination and soft contact lens fitting, as well as baseline measurements of refractive errors, IOP, and the anterior segment and retinal imaging. Refractive error and K readings were measured objectively using an autorefractor/keratometer (Nidek ARK-510A, Tokyo, Japan), and Medmont E300 Corneal Topography (Medmont Pty, Ltd., Melbourne, Australia) was used to choose the initial contact lens from the diagnostic set for each eye. A slit-lamp screening was performed to confirm their eligibility for the study based on our inclusion and exclusion criteria using a slit lamp biomicroscope (Carl Zeiss Meditec AG, Jena, Germany). During the screening visit, participants’ eligibility was determined, and baseline measurements, including subjective refraction, IOP (measured with a non-contact tonometer and a Diaton tonometer), retinal nerve fiber layer (RNFL) thickness, and blood pressure, were collected.

Contact lenses were randomly selected using the randomization generator and the participants’ sign-up order. The experiment was conducted on two separate days, ensuring a minimum washout period of 24 h between sessions to minimize carryover effects.

IOP and RNFL measurements were taken four times daily: (1) pre-lens application, (2) post-lens application, (3) pre-lens removal, and (4) post-lens removal. All pre- and post-lens application measurements were obtained in the morning, while pre- and post-lens removal measurements were taken in the evening. Each participant wore both lenses for 8 h. Figure 1 illustrates the data collection procedures.

The use of two different tonometers across measurement conditions was a deliberate design necessity rather than a methodological preference. Standard corneal tonometry, including non-contact air-puff tonometry, requires direct access to the corneal surface and cannot be performed while a scleral lens is in situ, as the lens completely vaults and covers the cornea. This fundamental constraint is well recognized in the scleral lens literature and has led investigators to adopt alternative approaches, including transpalpebral tonometry, peripheral scleral pneumotonometry, or post-removal corneal tonometry. The Diaton transpalpebral tonometer was selected for intra-wear measurements because it is currently one of the few devices available that can estimate IOP with the scleral lens in place and has been used in prior scleral lens studies for this purpose [20,21]. The NCT was retained for pre-lens application and post-lens removal measurements, as it provides more standardized and validated corneal IOP values at time points when the cornea is accessible.

Specifically, IOP measurements were obtained using a non-contact tonometer (Canon Full Auto Tonometer TX-F, Ota City Japan) for pre-lens application and post-lens removal. The Diaton tonometer (BICOM Inc., Long Beach, NY, USA) was applied to participants in a supine position, gazing at a target placed approximately 45 degrees downward. The probe was positioned vertically over the tarsus, just above the lens margin on the upper eyelid. At least three measurements were taken and averaged for the final IOP value at each time point. Any IOP readings falling outside ±2 mmHg of the first measurements were disregarded, and the average of three measurements was recorded as a single reading. IOP measurements were consistently obtained within 2–3 min after lens removal, a timeframe selected based on prior evidence indicating that post-lens IOP fluctuations occur rapidly within the first few minutes. Measurement consistency was enhanced by standardizing Diaton probe placement relative to the superior limbus, controlling upper eyelid positioning, utilizing a single experienced examiner for all measurements, and avoiding contact over the scleral lens landing zone. Although minor micro-variations in probe placement cannot be entirely eliminated, intersession variability remained low (standard deviation < 2 mmHg across repeated trials), supporting acceptable reproducibility. It must be acknowledged that IOP measurements obtained within 2–3 min of lens removal via NCT represent proxies for true intra-lens IOP rather than direct measurements. IOP normalization following scleral lens removal is known to occur rapidly, and values obtained within minutes of removal may already reflect partial recovery toward baseline rather than the peak IOP present during lens wear [19,21]. The concurrent Diaton transpalpebral measurements obtained during lens wear therefore serve as the more direct, albeit imperfect, estimate of IOP in situ.

Optovue optical coherence tomography (Optovue RTVue-100; Optovue Inc., Fremont, CA, USA) was used to measure RNFL thickness, with a scanning speed of 70,000 A-scans/second, optical axial resolution of 5 µm, and transverse resolution of 15 µm. The optic nerve head scan protocol consisted of four circular scans with a 3.45 mm diameter centered on the optic disc, each containing 1024 A-scans. RNFL measurements were taken at baseline and under all four conditions. Three scans were obtained and averaged to derive the final RNFL value at each time point. Peripapillary, overall average, superior, and inferior RNFL thickness were analyzed.

To ensure data quality, only scans meeting a minimum Signal Strength Index (SSI) of 40 or above were accepted for analysis, consistent with the manufacturer’s quality guidelines for the Optovue RTVue-100 system, in which SSI ≥ 40 represents adequate scan quality suitable for RNFL analysis [30]. Scans falling below this threshold or exhibiting segmentation errors, motion artifacts, or poor centration were immediately repeated. The instrument underwent its built-in automated calibration verification at the start of each study visit prior to data collection. All OCT acquisitions were performed in a standardized darkened examination room by a single trained operator to minimize inter-operator variability and ensure consistent ambient lighting and pupil state across all time points and visits. Measurements were conducted at the same time of day for each participant to minimize the influence of diurnal variation on RNFL thickness. The “average RNFL thickness” was determined as the automated average of superior and inferior RNFL thickness from the optic nerve head scan.

### 2.3. Randomization and Crossover Protocol

A prospective randomized contralateral-eye crossover design was employed. Participants were randomized using a computer-generated allocation sequence to determine which eye received the scleral lens (SL) and which received the soft contact lens (SCL) at Visit 1. Following a washout period of at least 24 h without lens wear, lens assignments were reversed between eyes at Visit 2. This design allowed each eye to serve as its own control while minimizing inter-subject variability.

Contact lens fitting was performed using a standardized diagnostic set. Scleral lenses (16.5 mm Ampleye; Boston, MA, USA; manufactured in Contamac Extra, Contamac Ltd., Essex, UK) were fitted targeting 200–300 µm of apical corneal clearance after settling. Soft contact lenses (14.0 mm diameter) were selected based on horizontal visible iris diameter and refractive error to ensure optimal vision, centration, and movement. All fits were evaluated by slit-lamp biomicroscopy to confirm adequate centration, absence of conjunctival blanching or impingement, and overall ocular surface compatibility. A single experienced investigator performed all lens applications and removals to minimize procedural variability. The specifications of the scleral and soft contact lenses used in this study are presented in Table 1. 

### 2.4. Statistical Analysis

With an effect size of 0.5 and a correlation of 0.7, sample size estimates were calculated for a power of 80% and a *p*-value of 0.05. The normality of the data was assessed using the Kolmogorov–Smirnov test. For each variable of interest (IOP, RNFL thickness, etc.), a linear mixed model (LMM) was used. Data were structured in long format with one observation per row, and participant ID was specified as a random effect to account for the non-independence of repeated measurements within the individual. Fixed effects included lens type (2 levels: scleral, soft), time point (4 levels), and their interaction (lens type × time point). Paired t-tests were also utilized for specific comparisons, with Bonferroni corrections applied to adjust for multiple comparisons where applicable. Bland–Altman analysis, linear regression, and the Pearson correlation coefficient were used to evaluate the performance of the two tonometers (Diaton and non-contact tonometer) in measuring IOP. All statistical analyses were performed using SPSS Statistics, version 28 (SPSS Inc., Chicago, IL, USA).

## 3. Results

### 3.1. Demographics and Clinical Characteristics

A total of thirty-one healthy participants (20 females, 64.5%), aged between 20 and 31 years (mean age 26 ± 3 years), were recruited and completed the study. The mean spherical equivalent was −3.25 ± 3.25 D, with a range of −9.25 to −0.50 D. All participants had normal baseline intraocular pressure (IOP), ranging from 8 to 20 mmHg, with a mean of 14.0 ± 3.0 mmHg. The demographic and clinical data of the participants are summarized in Table 2.

### 3.2. Intraocular Pressure (IOP)

To control for diurnal variation, all measurements for both IOP and RNFL thickness were taken at the same time of day for each participant during their experimental visits. The mean baseline IOP (pre-lens application), measured on the morning of the first experimental visit using the non-contact tonometer (NCT) and the Diaton tonometer, was 11.3 mmHg (95% CI, 11 to 12 mmHg). In contrast, the baseline IOP measured simultaneously with the NCT was 14.17 mmHg (95% CI: 14 to 15 mmHg). There was no significant difference in baseline IOP between the right and left eyes with either tonometer (all *p* > 0.05). The assumption of sphericity was tested using Mauchly’s test for IOP. The results indicated that the assumption of sphericity was met, with Mauchly’s test yielding a non-significant result (*p* = 0.48). The linear mixed model analysis revealed a significant main effect of lens type, F(1, 685) = 102, *p* < 0.001, and a significant main effect of time point, F(3, 685) = 13.3, *p* < 0.001. In addition, there was a significant interaction between lens type and time point, F(3, 685) = 31.7, *p* < 0.001.

**Figure 2:**
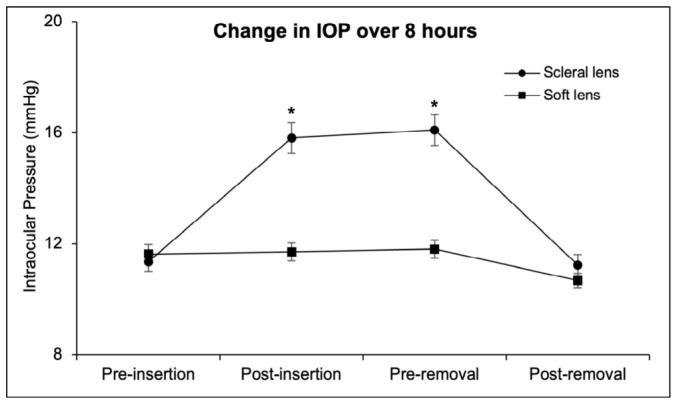
Mean intraocular pressure (IOP) measured by the Diaton tonometer at baseline, post-lens application, pre-lens removal, and post-lens removal in eyes wearing scleral lenses (SLs) and soft contact lenses (SCLs). Error bars represent 95% confidence intervals. * Indicates significant difference between lenses at the same time point (*p* < 0.05).

After lens application, IOP was measured using the Diaton tonometer within 5 min of lens wear. In eyes fitted with scleral lenses, the mean IOP increased to 15.8 mmHg (95% CI, 15 to 17 mmHg), representing a statistically significant rise of 4.5 mmHg from baseline (*p* < 0.0001), equivalent to approximately 40% above mean baseline IOP and corresponding to a very large effect size (Cohen’s d = 1.5). While this elevation is numerically consistent with a clinically meaningful change, the 3 mmHg threshold used in glaucoma clinical trials, and the poor agreement between the Diaton and NCT devices, as demonstrated by our Bland–Altman analysis, introduces substantial uncertainty in the absolute magnitude of this estimate. The true intra-lens IOP elevation may differ from the values reported, and these figures should be interpreted as approximate directional indicators rather than precise measurements [31]. In contrast, the mean IOP in eyes with soft contact lenses was 11.7 mmHg (95% CI, 11 to 12 mmHg), with no significant difference from baseline 11.63 mmHg (*p* = 0.48) (Figure 2).

During 8 h of contact lens wear, the average IOP in control eyes (soft contact lenses) remained stable at approximately 12 mmHg (95% CI, 11 to 12 mmHg) between post-lens application and pre-removal, with no significant change from application to removal (*p* = 0.63). There was no significant difference in IOP during the 8 h lens wear period (post-lens application and pre-removal) in the scleral lens group (*p* = 0.37) (Figure 2). The stability of IOP in control eyes throughout the 8 h wear period (*p* = 0.63) provides direct empirical evidence against a clinically meaningful effect on fellow-eye IOP during unilateral scleral lens wear in our cohort, supporting the validity of the contralateral-eye design.

After lens removal, the soft contact lens group had a mean IOP (Diaton) of 10.68 mmHg (95% CI, 10 to 11 mmHg), which was a small but statistically significant reduction of 1 mmHg from baseline (Diaton, 11.63 mmHg [95% CI, −1 to −1 mmHg]; *p* = 0.02). In the scleral lens group, mean IOP after removal returned to baseline (Diaton, 11.2 mmHg [95% CI, 11 to 12 mmHg]; *p* = 0.71) (Figure 2).

Comparing Diaton and NCT for IOP measurements at baseline, there was a significant difference (Diaton, 11.35 ± 3 mmHg [95% CI, 11 to 12 mmHg]; NCT, 14.17 ± 3 mmHg [95% CI, 14 to 15 mmHg]; *p* < 0.001). Bland–Altman analysis revealed poor agreement between the two instruments (regression slope = 22, R^2^ = 0.024, Y-intercept = 9.5), with wide limits of agreement indicating that the two devices cannot be used interchangeably for IOP estimation. This finding is consistent with published literature reporting poor agreement between transpalpebral and corneal tonometry methods [32]. The poor inter-device agreement has three specific implications for interpreting our IOP results. First, absolute IOP values obtained with the Diaton during lens wear should not be directly compared with NCT values obtained before and after lens wear, as systematic between-device differences may confound apparent changes. Second, Diaton measurements during lens wear are best interpreted as directional trend indicators reflecting relative IOP changes over time rather than as precise absolute IOP estimates. Third, the consistent directional elevation in IOP observed across both devices and participants strengthens confidence in the overall finding of increased IOP during scleral lens wear, despite uncertainty in the absolute value. Notably, the Diaton tonometer demonstrated a differential IOP response between the two lens conditions, detecting a statistically significant IOP elevation during scleral lens wear (*p* < 0.0001) while showing no significant change during soft contact lens wear (*p* = 0.48). This differential response provides internal validation of Diaton’s directional sensitivity in this study and supports the qualitative finding of scleral lens-induced IOP elevation, regardless of the scleral lens’s absolute measurement accuracy. A linear mixed model summary for IOP, showing both main effects and interactions, is provided in Table 3.

### 3.3. Retinal Nerve Fiber Layer (RNFL) Thickness

The mean overall RNFL thickness at baseline, measured in the morning, was 98.9 ± 7 μm (95% CI, 97 to 101 μm) in eyes fitted with scleral lenses and 98.6 ± 8 μm (95% CI, 97 to 101 μm) in eyes fitted with soft contact lenses (*p* = 0.23). The assumption of sphericity was tested using Mauchly’s test for RNFL. The results indicated that the assumption of sphericity was met, with Mauchly’s test yielding a non-significant result (*p* = 0.62). For the overall RNFL, the linear mixed model analysis showed a significant main effect of lens type, F(1, 701) = 18, *p* < 0.001, and a significant main effect of time point, F(3, 701) = 47, *p* < 0.001. In addition, there was a significant interaction between contact lens type and time point, F(3, 701) = 13.5, *p* < 0.001.

After lens application, eyes fitted with scleral lenses showed a reduction in overall RNFL thickness, reaching a mean of 96 ± 8 μm (95% CI, 95 to 98 μm) (*p* < 0.001). In contrast, eyes fitted with soft lenses showed unchanged mean thickness of 98 μm (95% CI, 97 to 100 μm) (*p* < 0.30). Prior to lens removal, eyes fitted with scleral lenses showed a reduction in overall RNFL thickness, reaching a mean of 96 ± 8 μm (95% CI, 95 to 98; *p* < 0.001). Eyes fitted with soft lenses did not exhibit a statistically significant reduction in mean thickness, with a value of 98 ± 8 μm (95% CI, 97 to 100 μm; *p* < 0.11). suggesting that the initial thinning observed with scleral lenses occurred shortly after lens application and was maintained throughout the wear period.

Following lens removal, the overall RNFL thickness returned toward baseline in both the scleral lens group (97.8 ± 8 μm [95% CI, 96 to 100 μm]; *p* = 0.07) and the soft lens group (97.6 ± 8 μm [95% CI, 96 to 100 μm]; *p* = 0.10). Over the 8 h lens wear period, overall RNFL thickness remained stable in both the scleral and soft lens groups (*p* = 0.27 and 0.1, respectively). Following lens removal, the overall RNFL thickness returned to baseline in both the scleral lens group (97.8 μm [95% CI, 96 to 100 μm]; *p* = 0.07) and the soft lens group (97.6 μm [95% CI, 96 to 100 μm]; *p* = 0.1). The linear mixed model summary for RNFL, showing both main effects and interactions, is provided in Table 4.

Although both lens types were associated with transient RNFL thinning, only the scleral lens group demonstrated concurrent IOP elevation, suggesting that different mechanisms, such as mechanical deformation, fluid dynamics, or measurement artifact, may underlie these changes. This distinction warrants further investigation and is discussed in more detail below.

**Figure 3:**
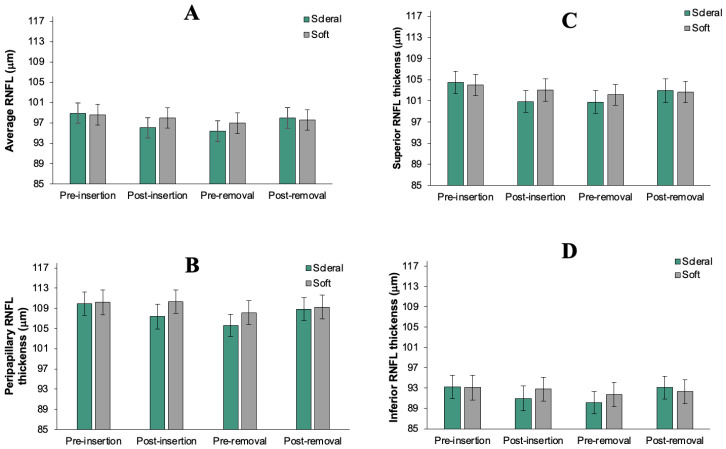
Mean retinal nerve fiber layer (RNFL) thickness (µm) measured by optical coherence tomography at baseline, post-lens application, pre-lens removal, and post-lens removal in eyes wearing scleral lenses (SL) and soft contact lenses (SCL). Panels show average (**A**), peripapillary (**B**), superior (**C**), and inferior (**D**) RNFL thickness. Error bars represent 95% confidence intervals.

Further analysis of the peripapillary, superior, and inferior RNFL thickness revealed no significant differences in peripapillary RNFL (*p* = 0.41), superior RNFL (*p* = 0.20), or inferior RNFL (*p* = 0.74) before lens application between the scleral and soft lens groups. However, consistent with the observed overall RNFL thickness reduction after scleral lens application, significant thinning was observed in peripapillary (*p* < 0.001), superior (*p* < 0.001), and inferior RNFL thickness (*p* < 0.001). The most pronounced thinning occurred in the peripapillary region (mean difference from baseline = −2.9 μm), followed by superior RNFL (−2.2 μm), average RNFL (−1.9 μm), and inferior RNFL (−1.8 μm) thickness. There were no significant differences in RNFL thickness between pre-lens application and post-lens removal (Figure 3A–D).

To explore the mechanistic relationship between scleral lens-induced IOP elevation and RNFL changes, a Spearman correlation analysis was performed between the magnitude of IOP change (ΔIOP, Diaton) and the corresponding average RNFL change (ΔRNFL) at the post-lens application time point across all 31 participants. Given that ΔIOP was normally distributed (Shapiro–Wilk W = 0.948, *p* = 0.142) while ΔRNFL was not (W = 0.809, *p* < 0.001), Spearman correlation was used. No statistically significant correlation was observed between ΔIOP and ΔRNFL (ρ = 0.071, *p* = 0.706, 95% CI −0.291 to 0.415, *n* = 31).

## 4. Discussion

The present study employed a prospective randomized crossover design, which provides strong within-subject control but does not permit causal inference. The temporal associations between scleral lens wear, IOP elevation, and RNFL thinning reported below should therefore be interpreted as observational findings rather than evidence of direct causation.

This study investigated the short-term effects of scleral lens wear on IOP and RNFL thickness in healthy young adults with a wide range of refractive errors. A significant increase in IOP, averaging 4 mmHg, was observed during scleral lens wear and persisted until lens removal. Simultaneously, a statistically significant thinning of the RNFL was detected, suggesting a temporal association between the observed RNFL changes and IOP elevation during lens wear.

Following scleral lens removal, IOP in the test eye dropped significantly to baseline levels, with less than a 1 mmHg difference between test and control eyes using the Diaton tonometer. However, it should be noted that post-removal NCT measurements are proxies for true intra-lens IOP rather than direct measurements, as rapid IOP normalization following lens removal means these values may already reflect partial recovery toward baseline rather than peak intra-lens pressure [19]. These findings align with previous studies reporting transient increases in IOP during scleral lens wear. Proposed mechanisms for this IOP elevation include compression of the bulbar conjunctiva, potentially altering aqueous humor dynamics, increased red cell content, and local cyanosis in the scleral lens-covered region [33]. Additionally, post-lens fluid forces and eyelid tension may contribute to altered lens fit and compression of conjunctival and scleral tissues, potentially contributing to IOP elevation [22]. A previous study reported an average IOP increase of 5 mmHg with a 16.5 mm diameter scleral lens, consistent with prior research reporting similar IOP elevations regardless of lens diameter [20]. However, due to the lack of a standardized device for measuring IOP during scleral lens wear, the clinical significance of these findings remains inconclusive.

The poor agreement between Diaton tonometer and NCT measurements highlights the variability and limitations of current methods for assessing IOP in the presence of scleral lenses [32,34]. While the NCT showed better agreement with GAT, inconsistencies in Diaton measurements underscore the need for improved measurement techniques in such scenarios [21].

Changes in RNFL thickness during scleral lens wear were also explored. To our knowledge, this is the first study to directly examine peripapillary RNFL thickness during extended scleral lens wear (8 h) in healthy adults, using a randomized contralateral-eye crossover design with a soft-lens control condition. The RNFL thinning observed immediately after scleral lens application was temporally coincident with the IOP elevation; however, the absence of a statistically significant IOP–RNFL correlation (ρ = 0.071, *p* = 0.706) substantially weakens the argument that the observed RNFL changes were driven by IOP elevation. Alternative explanations must therefore be considered as primary candidates. The clinical interpretation of the observed RNFL thinning requires careful consideration of OCT measurement variability. Published test–retest variability for average RNFL thickness measured with the Optovue RTVue-100 ranges from approximately 4.72 µm in healthy subjects [35]. The observed mean thinning of 1.8–2.9 µm across RNFL subfields, therefore, falls below the lower boundary of published average RNFL test–retest variability for this device. These findings should not be interpreted as unequivocal evidence of true structural change at the individual level. While the statistical significance reflects a consistent directional trend at the group level, this does not confirm that genuine structural alterations have occurred. Given that the observed thinning falls below the instrument’s test–retest variability threshold, these findings should be regarded as preliminary and hypothesis-generating rather than confirmatory of true structural change. Larger longitudinal studies with multiple repeated OCT measurements per time point are needed to determine whether scleral lens wear produces genuine cumulative RNFL changes beyond measurement noise.

The RNFL result aligns with previous studies indicating that RNFL thickness correlates with other structural metrics, such as minimum rim width [36]. Additionally, RNFL thinning throughout the day is well-documented, as diurnal variations lead to greater thinning in the afternoon and evening compared to morning measurements [37]. However, the RNFL thinning detected in this study occurred shortly after lens application, which may be consistent with an immediate response to mechanical or pressure-related factors, though causality cannot be confirmed. Several alternative explanations for the observed RNFL thinning must be considered. First, diurnal variation in RNFL thickness is a well-recognized phenomenon, with studies demonstrating progressive thinning throughout the day in healthy subjects [38]. In the present study, measurements were taken at standardized time points within a single session, and the crossover design means any consistent diurnal decline would affect both lens conditions equally, partially controlling for this confounder. However, a contribution from diurnal variation cannot be entirely excluded. Second, scleral lens wear is known to induce mild central corneal edema, which may reduce OCT signal quality and produce artifactual RNFL thinning [35]. While all scans met the minimum SSI threshold of ≥40, subtle signal degradation within the acceptable range may still have contributed to apparent thinning. Future studies should report SSI values at each time point to allow direct assessment of this potential artifact. Third, variability in OCT segmentation algorithms may contribute to apparent differences in RNFL thickness across time points, particularly in the context of changing anterior segment conditions during scleral lens wear [39]. Additionally, the optical properties of the scleral lens may introduce subtle changes in OCT scan magnification and signal quality, potentially contributing to apparent RNFL thickness differences; however, prior studies suggest that contact lens power does not significantly affect global RNFL thickness measurements [40]. Fourth, scleral lens-induced IOP elevation and episcleral vascular compression may transiently reduce peripapillary blood flow, producing apparent RNFL thinning through vascular rather than purely mechanical mechanisms [41]. This vascular hypothesis cannot be distinguished from a mechanical IOP-stress hypothesis on the basis of the present data alone and represents an important avenue for future investigation using OCT-angiography.

The natural homeostasis of IOP involves complex regulatory mechanisms that help mitigate transient IOP spikes, such as those observed during scleral lens wear. Factors such as trabecular meshwork adaptation, enhanced pulsatile flow, or reduced upstream resistance play a critical role in maintaining stable IOP [42]. In healthy individuals, these mechanisms are generally sufficient to prevent prolonged stress on the optic nerve head. However, glaucomatous eyes, which lack robust autoregulatory mechanisms, may potentially increase their susceptibility to IOP-related stress [13,42]. Future studies should replicate similar experiments in glaucoma patients to assess whether scleral lens wear poses additional risks in this population. While scleral lenses are traditionally used to manage corneal ectasias and surface irregularities, their growing adoption among individuals with high myopia highlights the importance of understanding their potential impacts on IOP and ocular health. Elevated IOP may increase susceptibility to glaucoma-like structural changes in highly myopic eyes due to the unique biomechanical and structural features of high myopia [15]. Axial elongation, a hallmark of high myopia, is associated with stretching and thinning of the sclera [16]. This thinning may reduce the sclera’s ability to provide structural support to the ONH and surrounding tissues, potentially increasing susceptibility to IOP-related stress [43]. Additionally, scleral thinning is often accompanied by changes in scleral stiffness; the sclera becomes more compliant and less able to resist deformation under elevated IOP. This increased biomechanical susceptibility may exacerbate the transmission of stress and strain to the ONH, potentially contributing to deformation of the lamina cribrosa and disruption of axonal pathways [44]. Furthermore, in highly myopic eyes, the elongated geometry creates a steeper translaminar pressure gradient, further amplifying the mechanical impact of elevated IOP on the ONH [15]. These combined factors, axial elongation, scleral thinning, and altered stiffness, may render highly myopic eyes less capable of withstanding IOP fluctuations, potentially increasing susceptibility to glaucomatous damage even at IOP levels considered normal in non-myopic eyes [45]. These considerations suggest that clinicians may wish to exercise heightened vigilance regarding IOP and posterior segment changes in highly myopic patients using scleral lenses, pending formal evidence-based guidance in this area. For future studies, vascular landmarks should be integrated into the analysis to better quantify potential variations in transverse magnification and to realign images, ensuring direct comparison of RNFL thickness at the exact same location. It should be noted, however, that these considerations are theoretical and require direct investigation in at-risk populations before any clinical recommendations can be made.

## 5. Conclusions

To our knowledge, this is the first study to evaluate peripapillary RNFL thickness as a structural outcome during extended scleral lens wear in healthy adults, using a randomized contralateral-eye crossover design with a soft lens control condition. The findings demonstrate a statistically significant increase in IOP in healthy eyes during scleral lens use, accompanied by a small but statistically consistent directional trend toward peripapillary RNFL thinning. However, the observed RNFL changes fell below published OCT test–retest variability thresholds and cannot be interpreted as confirmed structural change. These findings are exploratory and hypothesis-generating rather than confirmatory, and causality cannot be established from the present observational design.

These initial results warrant further investigation in larger, more diverse populations with extended wear durations to determine whether cumulative or long-term exposure may produce clinically significant structural or functional consequences. This is particularly relevant for individuals at higher risk of optic nerve vulnerability, such as those with high myopia or other predisposing conditions. Future longitudinal studies in clinically relevant populations will be needed before evidence-based clinical guidelines regarding IOP and RNFL monitoring during scleral lens wear can be established.

## 6. Limitations

This study was limited by its short-term evaluation of SCL wear (eight hours) in healthy young adults, which may not reflect the long-term effects on IOP and RNFL thickness. Healthy eyes may temporarily regulate IOP increases, but the duration and extent of this compensation remain unclear. Furthermore, the exclusive recruitment of healthy adults limits the generalizability of our findings to the populations most commonly prescribed scleral lenses in clinical practice, including those with keratoconus, corneal ectasia, and ocular surface disease. These populations may exhibit altered baseline IOP regulation, compromised corneal biomechanics, and pre-existing structural vulnerability of the optic nerve, all of which could amplify or modify the ocular responses observed in our healthy cohort [15,16]. Future studies should specifically investigate scleral lens-induced IOP and RNFL changes in these clinically relevant groups, particularly given the recent evidence that keratoconus patients may show BMO-MRW changes during scleral lens wear [10]. Additionally, glaucomatous eyes and individuals with collagen disorders like keratoconus may respond differently due to impaired IOP regulation, underscoring the need for further research in these populations.

A further methodological limitation is the need to use two different tonometers across measurement conditions. While the NCT provided standardized corneal IOP measurements at baseline and post-removal, the Diaton transpalpebral tonometer was required for intra-wear measurements, given the inaccessibility of the cornea during scleral lens wear. The poor agreement between these devices, as demonstrated by our Bland–Altman analysis, limits direct cross-condition comparisons of IOP and introduces uncertainty in the absolute magnitude of IOP change during lens wear. This is an inherent challenge shared by all scleral lens IOP studies to date and reflects the current absence of a validated gold-standard method for measuring true IOP during scleral lens wear [19,21].

The absence of Goldmann applanation tonometry (GAT) from the study protocol represents a further limitation. GAT was not feasible as corneal applanation cannot be performed with a scleral lens in situ. NCT was used as a validated surrogate, with published evidence demonstrating good NCT–GAT agreement in healthy subjects [46]. However, the absence of GAT introduces a degree of measurement imprecision relative to the gold standard that should be considered when interpreting the absolute IOP values reported.

Additionally, IOP measurements obtained within 2–3 min of lens removal via NCT should be interpreted as proxies for true intra-lens IOP rather than direct measurements of peak intra-lens pressure. Given the documented rapidity of IOP normalization following scleral lens removal, these post-removal values likely underestimate the peak IOP elevation present during lens wear [19,21]. The concurrent Diaton transpalpebral measurements obtained during lens wear therefore serve as the more direct, albeit imperfect, estimate of IOP in situ. Future studies should prioritize validated intra-wear tonometry methods to more accurately capture true IOP during lens wear.

The contralateral-eye design carries a theoretical risk that consensual inter-eye interactions could influence control-eye measurements, as fellow-eye responses have been documented in contact lens studies [47]. However, IOP in control eyes remained stable throughout the 8 h wear period (*p* = 0.63), providing direct empirical evidence against a clinically meaningful fellow eye effect in our cohort. Additionally, the repeated-measures crossover design ensures that any consistent fellow-eye effect would be absorbed into the within-subject baseline and would not inflate the reported between-condition differences.

The sample size of 31 participants, while adequate for the primary IOP outcome and consistent with prior scleral lens studies, represents a potential limitation for the RNFL secondary outcome. Post hoc power analysis suggests the study had approximately 75–80% power to detect the observed mean RNFL thinning of 2–3 µm in the repeated-measures crossover design. The study is underpowered for detecting smaller RNFL changes with high confidence. Future studies should be specifically powered for RNFL detection with a minimum of 40–50 participants and should incorporate multiple repeated OCT measurements per time point to better characterize true structural change versus measurement noise.

Disclosure: This research was conducted as partial fulfillment of the requirements for the Master of Science in Vision Science degree at Pacific University School of Optometry. The manuscript/abstract was previously submitted as a thesis and deposited in the Pacific University Library repository.

## Figures and Tables

**Table 1:** Specifications of Scleral and Soft Contact Lenses.

	Scleral Lens	Soft Lens
Contact lens brand	Comfort Optimum Extra	Acuvue Oasys 1 Day
Material	Roflufocon D	Senofilcon A
Refractive index	1.43	1.40
Dk value (Barrer)	100	103
Diameter (mm)	16.5	14.3

**Table 2:** Demographics and clinical characteristics of Participants.

	N (%)	Mean ± SD	Range
Age(years)		26 ± 3	20–31
Sex, male/female: *n* (%)	20/11 (64.5%/35.5%)		
Race: *n* (%)			
White	18 (58.1%)		
Asian	3 (9.7%)		
Asian/Indian	3 (9.7%)		
Other	7 (22.6%)		
Refractive error (Spherical equivalent) (D)		−3.25 ± 3.25	−9.25–+0.50
Average corneal curvature (D)		43 ± 2.25	40.25–46.5
Baseline IOP (mmHg)		14.3 ± 3	8–20
Pulse (BPM)		72 ± 13	46–97
Systolic BP (mmHg)		105 ± 13	84–140
Diastolic BP (mmHg)		62 ± 8	43–86

**Table 3:** Linear mixed model summary table for IOP showing both main effects and interactions.

Source	df (Num, Den)	F	Sig.	Cohen’s d
Lens type	1, 685	102.13	<0.001	1.16
Time point	3, 685	13.34	<0.001	-
Lens type X Time point	3, 685.211	31.741	<0.001	-

Cohen’s d reported for the primary between-condition comparison (scleral vs. soft lens) at the post-application time point.

**Table 4:** Linear mixed model summary table for RNFL showing both main effects and interactions.

Source	df (Num, Den)	F	Sig.	Cohen’s d
Lens type	1, 701	17.925	<0.001	−0.24
Time point	3, 701	47.057	<0.001	-
Lens type X Time point	3, 701	13.552	<0.001	-

Cohen’s d reported for the primary between-condition comparison (scleral vs. soft lens) at the post-application time point.

## Data Availability

The datasets generated and/or analyzed during the current study are available from the corresponding author upon reasonable request. The data are not publicly available because they contain information from human participants and are subject to institutional ethical and privacy restrictions.
